# Supplementary material for: Causal Relationship between Aging and Anorexia Nervosa: A White-Matter-Microstructure-Mediated Mendelian Randomization Analysis
Source: Biomedicines. 2024 Aug 16;12(8):1874. doi: 10.3390/biomedicines12081874 (PMC11351342; doi:10.3390/biomedicines12081874)
Supplement: Supplementary file 1 [file biomedicines-12-01874-s001.zip › Supplementary Figure/Figure S4/Singe SNP plot of MR analysis for TL to ubm-b-1487.pdf]

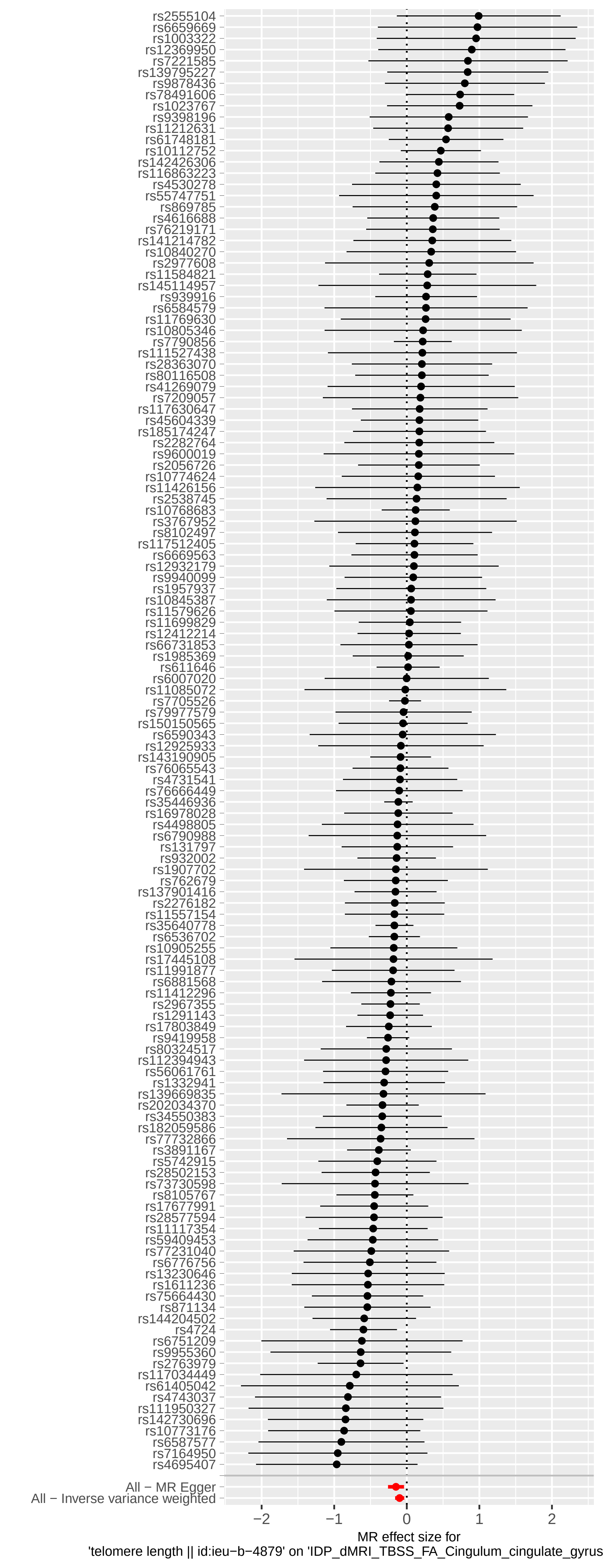
rs2555104
rs6659669
rs1003322
rs12369950
rs7221585
rs139795227
rs9878436
rs78491606
rs1023767
rs9398196
rs11212631
rs61748181
rs10112752
rs142426306
rs116863223
rs4530278
rs55747751
rs869785
rs4616688
rs76219171
rs141214782
rs10840270
rs2977608
rs11584821
rs145114957
rs939916
rs6584579
rs11769630
rs10805346
rs7790856
rs111527438
rs28363070
rs80116508
rs41269079
rs7209057
rs117630647
rs45604339
rs185174247
rs2282764
rs9600019
rs2056726
rs10774624
rs11426156
rs2538745
rs10768683
rs3767952
rs8102497
rs117512405
rs6669563
rs12932179
rs9940099
rs1957937
rs10845387
rs11579626
rs11699829
rs12412214
rs66731853
rs1985369
rs611646
rs6007020
rs11085072
rs7705526
rs79977579
rs150150565
rs6590343
rs12925933
rs143190905
rs76065543
rs4731541
rs76666449
rs35446936
rs16978028
rs4498805
rs6790988
rs131797
rs932002
rs1907702
rs762679
rs137901416
rs2276182
rs11557154
rs35640778
rs6536702
rs10905255
rs17445108
rs11991877
rs6881568
rs11412296
rs2967355
rs1291143
rs17803849
rs9419958
rs80324517
rs112394943
rs56061761
rs1332941
rs139669835
rs202034370
rs34550383
rs182059586
rs77732866
rs3891167
rs5742915
rs28502153
rs73730598
rs8105767
rs17677991
rs28577594
rs11117354
rs59409453
rs77231040
rs6776756
rs13230646
rs1611236
rs75664430
rs871134
rs144204502
rs4724
rs6751209
rs9955360
rs2763979
rs117034449
rs61405042
rs4743037
rs111950327
rs142730696
rs10773176
rs6587577
rs7164950
rs4695407
All − MR Egger
All − Inverse variance weighted
−2
−1
0
1
2
MR effect size for
'telomere length || id:ieu−b−4879' on 'IDP_dMRI_TBSS_FA_Cingulum_cingulate_gyrus
